# Integrating Small Animal Irradiators with Functional Imaging for Advanced Preclinical Radiotherapy Research

**DOI:** 10.3390/cancers11020170

**Published:** 2019-02-01

**Authors:** Mihaela Ghita, Kathryn H. Brown, Olivia J. Kelada, Edward E. Graves, Karl T. Butterworth

**Affiliations:** 1 Centre for Cancer Research and Cell Biology, Queen’s University Belfast, Belfast BT9 7AE, Northern Ireland, UK; m.ghita@qub.ac.uk (M.G.); kbrown40@qub.ac.uk (K.H.B.); 2 Molecular Imaging Program, National Cancer Institute, National Institutes of Health, Bethesda, MD 20892-1088, USA; olivia.kelada@nih.gov; 3 In vivo Imaging, Discovery and Analytics, PerkinElmer Inc., Hopkinton, MA 01748, USA; 4 Department of Radiation Oncology, Stanford University, Stanford, CA 94305-5152, USA; egraves@stanford.edu

**Keywords:** preclinical radiotherapy, functional imaging, small animal irradiators, radiobiology, radiation oncology

## Abstract

Translational research aims to provide direct support for advancing novel treatment approaches in oncology towards improving patient outcomes. Preclinical studies have a central role in this process and the ability to accurately model biological and physical aspects of the clinical scenario in radiation oncology is critical to translational success. The use of small animal irradiators with disease relevant mouse models and advanced in vivo imaging approaches offers unique possibilities to interrogate the radiotherapy response of tumors and normal tissues with high potential to translate to improvements in clinical outcomes. The present review highlights the current technology and applications of small animal irradiators, and explores how these can be combined with molecular and functional imaging in advanced preclinical radiotherapy research.

## 1. Introduction

Since the introduction of the linear accelerator into the practice of radiation oncology during the 1950s, the discipline has undergone major technology changes that have significantly advanced all stages of the radiotherapy process from treatment planning to delivery and verification. These innovations have resulted in an unparalleled ability to delineate target volumes, conform radiation dose and irradiate under image guidance [1], which have translated to better tumor control and reduced toxicity in many cancer types. Despite these advances, it is unlikely that radiotherapy technology has reached its zenith, with many developments in molecular and functional imaging, treatment adaptation and particle therapy yet to be fully realized in the clinic [2]. 

In contrast, the impact of biologically driven strategies in radiation oncology has been less substantial. This is evidenced by the implementation of most advanced radiotherapy techniques on the basis of technology rather than a comprehensive understanding of radiobiological response, highlighting the need for advanced preclinical systems capable of modelling aspects of human disease under clinically relevant radiation exposure conditions. In addition, several radiotherapy clinical trials have reported null outcomes, an issue that was examined by the National Cancer Institute (NCI) Radiation Research Program (RRP) at a workshop aiming to better understand these findings and to try to improve the success of future trials [3].

From radiotherapy trials reporting negative and null outcomes, an intriguing example is that of the phase 3 Radiotherapy Oncology Trial Group (RTOG) 0617 study. This aimed to compare standard-dose versus dose escalation with concurrent chemotherapy and the addition of cetuximab in patients with inoperable stage III non-small-cell lung cancer (NSCLC). The study failed to demonstrate overall survival benefit at the higher dose of 74 Gy, compared with the lower, standard dose of 60 Gy [4], and further reported 17 deaths in the high dose arms compared to 7 in the lower dose cohort. The causes of these unexpected findings have been explored with secondary analysis suggesting that deaths related to the effects of dose to the heart and lung are the most likely explanation of the findings, and these continue to be discussed [5].

Importantly, trials such as a RTOG 0617 need to be reverse translated using relevant preclinical models to gain de novo mechanistic insight into the clinical benefits and risks of dose escalation. Part of the recommendations proposed by the NCI RRP group have included the requirement for robust preclinical supporting data to guide subsequent clinical trials. In addition, Stone et al., surveyed data from 125 published reports which tested the interaction of 10 drug-radiation combinations and provided comprehensive recommendations for improved preclinical testing [6]. This has also been supported by further recommendations from Coleman et al., aiming at improve the predictive power of preclinical models in developing radiotherapy clinical trials [7]. Cumulatively, these reports clearly highlight the need for robust preclinical supporting data in translationally relevant disease models to justify radiotherapy clinical trials. In this context, it is essential that preclinical models in radiobiology research accurately reflect modern clinical practice, in terms of both biological model and physical radiation exposure conditions [8]. These approaches should also be further synergized with anatomical, functional and molecular imaging to optimize radiotherapy planning and response monitoring and maximize potential for translation. In this article, we review the technology of small animal irradiators and preclinical imaging techniques to identify key opportunities for translational research that may impact the future success rate of radiotherapy clinical trials.

## 2. Small Animal Radiotherapy: Rationale and Technology

Since the first report of the tissue sparing effects from fractionation in ram testes more than 100 years ago [9], small animal models have been widely applied in radiobiological studies predicated on the basis of genetic and physiological similarities with humans [10]. In particular, mouse models have contributed significantly to the advancement of biomedical research [11]. Recent genome editing technologies continue to allow a wide spectrum of gain- and loss-of-function mutations to be investigated along with evaluation of novel therapies in defined genomic backgrounds. In addition, the implantation of human tissue into NOD-scid-γ (NSG) mice with partially reconstituted immune systems can help to recapitulate aspects of the patient immune response during treatment through humanized mouse models [12]. Whilst no ideal mouse model exists to truly recapitulate the human setting, it is important that contemporary disease models are used with advanced irradiation techniques to closely mimic clinical scenarios and maximize the potential to deliver translationally relevant datasets [13].

**Figure 1:**
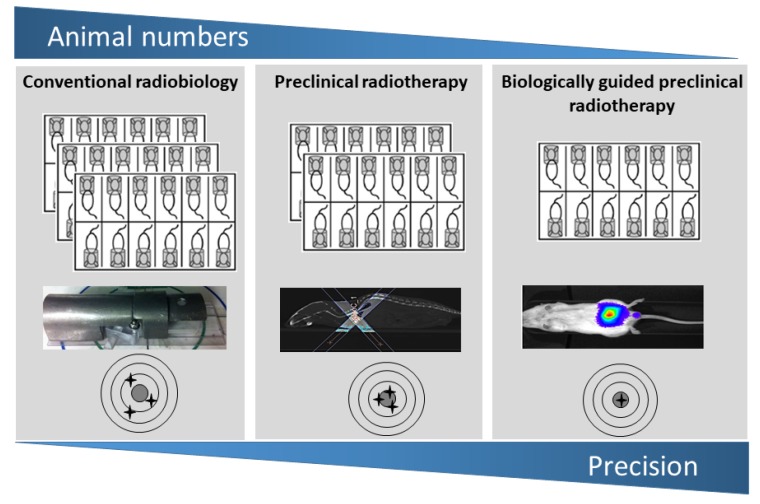
Schematic diagram demonstrating the evolution of conventional radiobiology to image-guided preclinical radiotherapy and molecular imaging. These changes are a major refinement of conventional techniques and have resulted in improved precision and accuracy. Overall, these advanced approaches have reduced study sizes in radiobiology studies required to obtain statistical power by reducing dose uncertainty, error and allowing longitudinal analysis.

Classic radiobiology experiments have been performed using broad fields from fixed sources and shielding to target the beam. Some of these experiments involved the irradiation of large volumes (usually whole body or whole thorax) which did not require precision image guidance, however, these procedures have high levels of uncertainty due to inaccurate beam targeting, as highlighted in Figure 1.

Similar to clinical techniques, modern radiobiology studies aim to irradiate small target volumes with high levels of precision and accuracy. Devices capable of performing image-guided irradiation in small animals have been developed over the last 10 years by a number of investigators and vendors (Table 1). Two systems have been made commercially available: The Small Animal Radiotherapy Research Platform (SARRP) from Xstrahl Life Sciences developed at Johns Hopkins University [13] and the X-Rad SmART from Precision X-ray Inc., developed at Princess Margaret Hospital [14]. In addition, several other systems have been designed and implemented at a number of institutions across the world using approaches consisting of rotating or fixed gantries with cone beam computed tomography (CBCT) detectors or conversions of micro-CT devices. Details of these systems and their characteristics are summarized in Table 1.

To complement these platforms, dedicated treatment planning systems (TPS) analogous to those used clinically are required for accurate dose calculations. These have been developed using the superposition convolution algorithm [25], Monte Carlo simulations [15] or in-house dose engines [26]. Preclinical TPS aim to efficiently solve the challenges associated with tissue segmentation and dose calculation relating to photon scattering for very small fields and differences in the energy absorption of soft tissues for kilovoltage beams [27]. Although still at a relatively early stage, small animal irradiators are rapidly becoming the experimental standard in radiobiology. Previously unachievable experimental approaches are now being explored, including the irradiation of small target volumes (under 100 mm^3^) at depth under image guidance from computed tomography (CT), magnetic resonance imaging (MRI) and bioluminescence imaging (BLI).

The integration of small animal irradiators into radiobiology research either, simultaneously or sequentially combined with imaging methods such as CT, BLI, positron-emission tomography (PET) and MRI, have significantly improved the level of precision and accuracy with which target volumes can be irradiated. These strategies have directly impacted animal welfare within the framework of the UK National Centre for the Replacement, Refinement and Reduction of Animals in Research (NC3Rs). In particular, these improvements are a major refinement from conventional approaches as both precision and accuracy has been improved along with the ability to acquire longitudinal information from the same animals. Consequently, this has reduced the requirement for large study sizes which shown schematically in Figure 1 [28,29].

## 3. Preclinical Imaging: Principles and Technology

Imaging has focused on the visualization of anatomical regions of interest in the diagnosis and staging of disease, as well as monitoring response to therapy. Conventional anatomical imaging methods, such as X-ray, fluoroscopy and computed tomography (CT) continue to play a critical role in the delineation of macroscopic patient anatomy. However, advances in molecular biology have enabled imaging techniques to move beyond structural characterization of malignancy into the realm of molecular imaging [30]. This involves the visualization, characterization, and measurement of biological processes at the molecular and cellular levels, typically including two- or three-dimensional imaging using techniques such as PET, Single Photon Emission Computed Tomography (SPECT) and BLI [31]. Some of these techniques overlap as functional imaging modalities that are used to delineate and measure physiologic functions in organ systems, including techniques such as PET, functional MRI and ultrasound.

**Figure 2:**
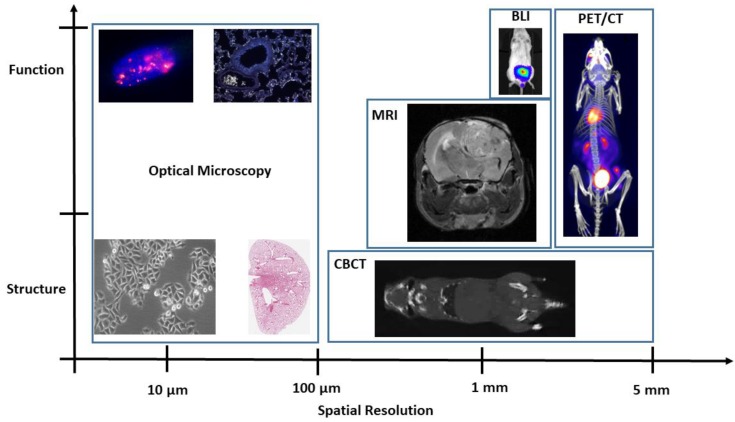
Schema of different preclinical imaging techniques showing increasing molecular specificity and spatial resolution for in vitro and in vivo studies. BLI, bioluminescence imaging; PET, positron-emission tomography; CT, computed tomography; MRI, magnetic resonance imaging.

Many preclinical imaging techniques were originally developed as clinical imaging procedures but have since been reverse translated in the same way modern radiotherapy has been brought to the preclinical laboratory. A variety of imaging methods are now available to plan and monitor radiotherapy response in a manner analogous to that in the clinic as shown in Figure 2.

X-ray computed tomography (CT) remains the primary imaging modality in preclinical and clinical radiotherapy treatment planning. In small animal CT scanners, X-rays are emitted as a beam from the tube, pass through the subject and are detected by a large-area solid-state radiation detector [28]. Whilst CT can provide material composition information useful for dose calculation, it has limited soft tissue contrast that can complicate identification of targets for treatment as well as volumes of radiosensitive tissues to be avoided.

A variety of molecular imaging technologies have been developed that generate image contrast based on functional aspects of tissue including perfusion, gene expression, oxygenation and metabolism, rather than anatomical structure [28,32,33,34]. The most prominent modality used for this purpose clinically is positron-emission tomography (PET), a nuclear medicine technique that detects and localizes radiation produced by positron-emitting radiopharmaceuticals administered exogenously to a subject [35]. Imaging glucose metabolism with ^18^F-fluorodeoxyglucose (^18^F-FDG) is most commonly used to provide functional information based on increased uptake and glycolysis of cancer cells [32,36]. ^18^F-FDG PET is widely used in cancer diagnosis and screening, yet it is unsuitable for tumors in organs with high ^18^F-FDG non-specific uptake such as the liver. Furthermore, it has limited ability to differentiate benign from metastatic lesions and early versus late stage disease [37].

The development of many other tracer types has given PET wide applications, particularly in the study of tumor metabolism [38]. Some preclinical PET tracers are probes consisting of a targeted molecule specific to a biological functional measurement attached to a radioisotope with a favorable half-life such as ^11^C, ^15^O and ^18^F. Probes can also be used to image specific molecules based on binding of radiolabeled ligands (e.g., α5β3 integrin imaging of tumor vasculature with radiolabeled glycosylated RGD (arginine-glycine-aspartate) containing peptides). A list of PET tracers used in preclinical studies and their biological targets is summarized in Table 2. PET has progressed to offer ~5 mm spatial resolution and picomolar sensitivity in clinical scanners [39]. Preclinical studies have exploited microPET technology with comparable sensitivity and ~1 mm spatial resolution for treatment planning and response assessment [28,40].

Magnetic resonance imaging (MRI) has become an established medical imaging modality due to its superior soft tissue contrast and lack of ionizing radiation dose. MRI is based on combining high-strength magnetic fields with radiofrequency (RF) detection to exploit atomic nuclei with odd numbers of nucleons and thus net magnetic moments [28]. At the microscopic level, net magnetic fields in tissue depend on the microenvironment as well as the applied magnetic field, yielding soft tissue contrast uniquely characteristic of MRI. Preclinical MRI systems are being implemented for a range of applications, including MRI-guided radiation therapy for intracranial, pancreatic and flank tumors, immobilization devices, fiducial marker placement and MRI-only based treatment planning [57,58,59]. These methods are well established for mice and rats with technical developments towards implementing adaptable registration into existing workflows for small animal radiotherapy [57,58,59].

Other molecular imaging modalities such as BLI have been developed specifically for preclinical applications. BLI involves the engineering of cells to express a luciferase enzyme, which catalyzes the metabolism of a substrate (luciferin) that generates as a by-product a photon in the visible to near-infrared wavelength range [49]. Such enzymatic reactions are found natively in organisms including bacteria, fireflies, and jellyfish, however, molecular biology methods have allowed integration of luciferase into cells in vitro or into animals through the germline. Two-dimensional BLI is now used extensively in preclinical cancer biology research to detect, quantify, and localize specific cell types [60,61,62] and is being integrated with preclinical image-guided irradiators for target localization and response monitoring.

The X-Rad SmART (Precision X-ray, Inc, North Branford, CT, USA) offers a configuration in which a cooled CCD camera is mounted on the gantry perpendicular to the X-ray beam axis to allow two-dimensional BLI data to be collected from the subject and co-registered with the planning CT [15]. In contrast, the SARRP offers MuriGlo, an optical imaging system capable of both two-dimensional BLI, fluorescence imaging and three-dimensional bioluminescence tomography (BLT) [63]. Similarly, BLT has also been successfully integrated into the iSMAART system and shown accurately targeting with quantitative assessment of response in orthotopically implanted, luciferase expressing 4T1 breast cancer cells [64]. BLI is an attractive imaging modality in radiotherapy studies as it can provide target localization information and does not contribute to added dose [65,66], however, research efforts are required to develop robust 3D reconstruction algorithms for true tomographical representation.

In addition to BLI, in vivo fluorescence imaging (FLI) is another optical imaging technique that is being applied to radiobiological studies. An interesting approach has been demonstrated combining the Cx225 platform with intra-vital, multimodal optical microscopy to study the spatio-temporal dynamics of tumor microvasculature in radiation response of tumors [67]. Another approach combined the iSMAART device with fluorescence molecular tomography (FMT) to localize tumors at depth with a localization error of <0.5 mm [19].

Finally, a number of imaging methods have been preclinically evaluated but have not been explored in the clinic. These include molecular ultrasound using microbubble agents, photoacoustic imaging, Raman spectroscopy, as well as a variety of emerging radiotracers, magnetic resonance imaging probes and X-ray contrast agents [28,34,46,68,69,70], which may yet provide important clinical advantages in diagnosis and treatment.

## 4. Translational Research Opportunities

Imaging techniques play a central role in patient management to determine tumor-specific characteristics and response to therapy. This is particularly evident in radiation oncology where imaging is used for diagnosis, treatment planning, response monitoring and to detect adverse effects resulting from treatment [35,71,72]. Preclinical investigations integrating anatomical, functional, and molecular imaging in the experimental study design allows interrogation of key molecular characteristics that can be used to detect and potentially predict radiotherapy response. CT and BLT have been successfully integrated with preclinical radiotherapy devices to provide excellent multi-modal imaging solutions. Using interchangeable beds and immobilization devices, it is also possible to develop sequential workflows across different systems allowing co-registration of multiple imaging sources with radiotherapy plans that are equally effective and more easily disseminated, e.g., sequential PET/MRI systems. Some of the unique research possibilities that these approaches are now enabling towards the realization of biologically optimized radiotherapy are explained below.

### 4.1. Quantifying Tumor Burden and Response to Therapy

Evaluation of the tumor burden at diagnosis and during response to therapy is critical in guiding treatment decisions, prognosis and radiotherapy planning. Clinically, longitudinal CT scans are used to assess volumetric differences in target lesions at baseline and after treatment, which are standardized to the Response Evaluation Criteria for Solid Tumors (RECIST) [73,74]. However, anatomical observations have limitations in imaging changes post treatment as they fail to accurately represent viable tumor cells. ^18^F-FDG-PET/CT has become established as an important tool in radiation oncology to determine primary tumor characteristics, lymph node invasion and metastases. This has led to the Positron Emission Tomography (PET) Response Criteria in Solid Tumors (PERCIST 1.0) which adopts functional imaging into routine assessment. These criteria also serve as a starting point for use in clinical trials when assessing the activity of novel therapies that stabilize disease, and has led to revised strategies based on functional rather than anatomical features [75].

Currently, no standardized staging or response criteria have been defined for preclinical studies where quantification of tumor burden is derived from caliper measurements or longitudinal monitoring of regions of interest delineated from imaging. In PET imaging, regions of interest are determined from the standardized uptake volume (SUV), defined as the level of tracer accumulation normalized to the subject mass. This may also be used to determine progression in cancer models by comparing tracer distributions in normal and diseased mice.

Preclinical efforts are needed towards optimizing PET tracers and evaluating radiotherapy response where determining success or failure may guide future treatment decisions such as dose boosting or salvage surgery. As tumor response is non-uniform, alterations in the SUVs of tracers often occurs prior to tissue changes and so may provide predictive information of response, local failure or radioresistance. Preclinical evaluation of ^18^F-FDG uptake has been determined for different doses and irradiated sub-volumes. This important study irradiated high uptake regions with high doses and reduced (redistribution approach) or standard doses (dose escalation approach) were delivered to the rest of the tumor volume. Minimum tumor growth delay was observed in the mice with dose escalation to the sub-volumes with high FDG uptake [47]. Other preclinical studies compare ^18^F-FMISO, ^18^F-FAZA and ^18^F-HX4 to optimize imaging conditions to evaluate tumor hypoxia in preclinical models [52,53,54]. A further study used the hypoxia specific tracer ^18^F-EF5 to determine changes in hypoxia with radiation response. Tracer uptake was correlated with tumor growth delay or total control and showed distinct responses corresponding to the uptake of ^18^F-EF5 before irradiation, suggesting response can be predicted based on initial ^18^F-EF5 uptake [49,50].

Finally, different imaging techniques can also allow differentiation of tumor and normal tissues at a level superior to anatomical imaging. Although technological improvements have reduced the risk of normal tissue injury, toxicity causing long-term side effects or interrupted treatment continues to occur in subsets of patients and can be critical in defining treatment options. Pre-clinically, CT data in free-breathing animals enabled the non-invasive and high-throughput detection of a range of pulmonary diseases in mice [76,77]. Moreover, CT was also used to image the radiation-induced lung fibrosis longitudinally for up to 9 months post irradiation [78,79,80,81]. MRI has also been used to detect pulmonary fibrosis in living mice and rats treated with bleomycin [82]. Considering the critical role of the immune system in radiotherapy response, imaging approaches capable of visualizing highly complex interactions involving multiple cell types are also being developed.

### 4.2. Imaging the Immune Response

The seminal discovery of enhanced antitumor immunity by blockade of the cytotoxic T-lymphocyte-associated protein 4 (CTLA-4) by Allison and colleagues in 1996 [83] has led to the rapid development and uptake of immunotherapy as standard of care for many types of cancer [84,85]. Currently, over 2000 different immunotherapy compounds are in development, with 26 approved immune-oncology (IO) agents already approved for use in the clinic including T-cell targeted immuno-modulators, cancer vaccines and cell therapies [85]. The underlying mechanism of action for IO agents often leads to abnormal response patterns termed pseudo-progression, and represents considerable risk in underestimating response which may lead to early removal of patients from treatment. This has resulted in the development of the iRECIST guidelines by the RECIST working group for the use of modified criteria in cancer immunotherapy trials [86], which provide information concerning tumor size but not biological characteristics of the tumor.

Given that only 15–30% of patients respond to IO agents as monotherapies, there is a critical need for biomarkers to accurately predict response and select patients most likely to respond, and for the development of novel combination therapies [87]. Radiotherapy is a promising approach for combination with IO due to its multiple immune-modulatory effects, which include natural killer (NK) cell activation [88], increased expression of tumor associated antigens [89] and activation of immunogenic cell death mediated by damage-associated molecular patterns (DAMPs) [87,90].

Limited information is available concerning the molecular imaging changes during response to IO agents as monotherapies or in combination with radiotherapy. Molecular imaging has much potential to differentiate immune response from tumor progression by targeted labelling of immune cells ex vivo, PET reporter gene expression or direct in vivo labelling and provides predictive biomarkers of response to IO in combination with radiotherapy [91]. Considering the observed dependence of immune effects on dose and fractionation scheme [92,93], major efforts are needed to optimize the combination of IO and radiotherapy in preclinical models prior to translation to the clinic. Important preclinical studies using syngeneic mouse mammary tumor models with small animal radiotherapy have delineated the mechanisms and dose response of radiation induced T cell activation through the DNA exonuclease, Trex1. These important findings may guide the selection of optimum radiation dose and fractionation in patients treated with immunotherapy [92]. Also, the tracer 2’-deoxy-2’-(^18^F)fluoro-9-β-D-arabinofuranosyl has been identified to have specific cytotoxicity in T-lymphocytes compared to other immune cell types and is currently under clinical investigation as an indicator of the immune status in cancer patients (NCT03142204). (^18^F)F-AraG has also been used in preclinical studies to image T-cell dynamics, showing up to 1.4-fold higher uptake in a Graft-versus-Host disease elicited by allogenic hematopoietic cell transplant compared to control mice [41,55,56].

### 4.3. Image-Guided Adaptive Radiotherapy

Anatomical changes during treatment including tumor growth, regression or weight loss may necessitate adaptive planning by modifying the treatment plan based on the most current image to prescribe new personalized margins and doses for individual patients [94]. Repeated CT imaging during treatment is often used for adaptive re-planning, aiming to integrate sequential imaging in the radiotherapy workflow. This has led to the development of MRI-integrated radiotherapy systems first conceptualized by Lagendijk and Bakker [95] with two systems now commercially available developed by ViewRay and the Philips Elekta Consortium [96,97].

In addition to real-time image-guided treatments, spatial variations in tracer uptake offer opportunities for dose boosting in sub-volumes of high ^18^F-FDG uptake or hypoxic regions [98,99,100,101]. Anatomical and functional imaging are proving essential in defining target volumes, in dose painting and adaptive treatments to optimize dose in radio-resistant areas, yet many challenges remain in how to best integrate these approaches and determine individualized treatments. Preclinical efforts have made towards developing novel technology, with several prototypes for MRI-PET imaging systems already developed and incorporated in pilot studies [101].

## 5. Conclusions

Technological innovations in the delivery of advanced conformal radiotherapy and radiological imaging have resulted in improved outcomes for cancer patients receiving radiotherapy. Clinical advances have been reverse translated to the laboratory, where research teams are now enabled to deliver highly conformal treatments to small volumes under image guidance. These advances fundamentally require a better understanding of the radiobiological correspondence between mice and humans so that fractionation schedules and dose distributions can be better interpolated in experimental models. The synergy of small animal radiotherapy studies with functional imaging has high potential to lead to the next generation of innovations in radiation oncology, which may include biologically guided treatments using predictive biomarkers to optimize dose, fractionation and combination treatments with IO or other molecular targeted agents.

## Figures and Tables

**Table 1:** Summary of small animal radiotherapy systems and individual characteristics.

Research Platform	Vendor Research Institute	Beam Energy (KeV)	Dose Rate (Gy/Min)	Beam Collimation	Accuracy (mm)	Image Guidance	Treatment Planning System	Reference
Commercially available								
SARRP ^1^	Xstrahl Life Sciences	5–225	1–4	Aperture MVC	0.2	CBCT BLT	Muriplan	[14,15]
X-RAD 225Cx SmART ^2^	Precision X-ray	5–225	0.01–4	Aperture	0.2	CBCT BLI	SmART-Plan	[16]
Non-commercial								
iSMAART	University of Miami, USA	45–225	2.5–4	Aperture	0.4	CBCT BLT FLT	In house	[17,18,19]
SAIGRT ^3^	Technical University of Dresden, Germany	10–225	1–4	Aperture MVC	0.1	CBCT	In house	[20]
SACRTD ^4^	University of Arkansas, AR, USA	60–225	0.4–3	Aperture	0.2	CBCT	In house	[21]
Micro-CT based radiotherapy devices	Stanford University, USA	70–120	2	Aperture	<0.1	CBCT	In house	[22]
	Heidelberg University, Germany	10–160	4.5–6.4	Aperture	<1	CBCT	In house	[23]
	The University of Western Ontario, Canada	70–140	2	Jaw Collimation	0.1	CBCT	In house	[24]

^1^ Small animal radiotherapy research platform (SARRP); ^2^ Small animal radiotherapy (SmART); ^3^ Small animal image guided radiotherapy (SAIGRT); ^4^ Small animal conformal radiotherapy device (SACRTD).

**Table 2:** Summary of tracers used in preclinical studies and their biological targets.

Tracer	Targeting Moiety	Biological Target	Reference
^64^Cu	Anti-PD-1	Tumor infiltrating lymphocytes	[41]
^124^I	Anti-CD4	CD8+ cells	[42,43,44]
^89^Zr	Anti-CD4	T-cell reconstitution post-transplant	[43]
^89^Zr	Anti-CD3	tumor-infiltrating lymphocytes	[44]
^64^Cu	Anti-OX40	T cells activation	[45]
	Anti-CTLA-4	CTLA-4 visualization	
^68^Ga/^18^F	PSMA	PSMA	[46]
^18^F-FDG	Fluorodeoxyglucose	Glucose metabolism	[47]
^68^Ga-NODAGA-c(RGDfK)	RGD (arginine, glycine, aspartate) peptides	αvβ3 integrins in the tumor vasculature	[48]
^18^F-EF5	2-(2-Nitro-1H-imidazol-1-yl)-N-(2,2,3,3,3-pentafluoro propyl)-acetamide	Hypoxia	[49,50]
^18^F-FAZA	1-(5-fluoro-5-deoxy-α-D-arabinofuranosyl)-2-nitroimidazole		
^18^F-FMISO	Fluoromisonidazole		[51,52,53,54]
^18^F-HX4	fluoro-2-(4-((2-nitro-1H-imidazol-1-yl)methyl)-1H-1,2,3-triazol-1-yl)propan-1-ol		
(^18^F)F-AraG	2-(2-Nitro-1H-imidazol-1-yl)-N-(2,2,3,3,3-pentafluoro propyl)-acetamide fluoro-9-β-D-arabinofuranosyl guanin	T cell activation	[55,56]
